# Targeting fibroblast growth factors in cancer

**DOI:** 10.18632/oncotarget.1054

**Published:** 2013-05-24

**Authors:** Kevin P. Baker, Gerrit Los

**Affiliations:** FivePrime Therapeutics, Inc., South San Francisco, CA, USA; FivePrime Therapeutics, Inc., South San Francisco, CA, USA

Fibroblast growth factors (FGFs) have been implicated in multiple aspects of cancer development and growth for over 20 years [1]. Research has shown that FGFs have a role in a) directly promoting cancer cell growth, b) tumor angiogenesis, and c) more speculatively, the survival of a group of cancer cells called “tumor stem cells” that are particularly difficult to target with current therapies. Therefore, inhibition of the FGF pathway has “multiple ways to win” as a cancer therapeutic.

The FGF family is large: it has 22 different ligand members (the FGFs) that bind to 4 different receptors (the FGFRs). FGFRs are type 1 transmembrane receptors containing a large extracellular domain that bind FGFs, a transmembrane domain and an intracellular signaling domain that contains a tyrosine kinase domain. Many ligands of the FGF family have been implicated in the initiation, promotion and maintenance of cancer. We term these cancer-related FGFs “the classical FGFs”. They play essential roles during normal embryonic development, but their roles are less important during normal adult life. During cancer development some tumors hijack the FGF pathway, thereby increasing the levels of expression of many FGF family members to promote growth and tumor metastasis.

A sub-group of the FGF family termed the hormonal FGFs has a more homeostatic role in the adult that includes regulating phosphate levels, vitamin D metabolism (FGF-23), bile acid production (FGF-19) and glucose utilization (FGF-21). Hormonal FGFs require co-receptors such and α-Klotho or β-Klotho to bind and signal via FGFRs [2].

**Figure 1:**
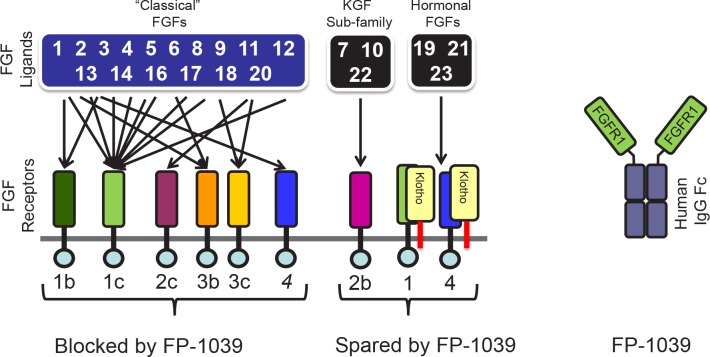
The family of FGF receptors and FGF ligands

FP-1039 (also known as HGS1036 or GSK3052230) is designed around FGF receptor 1 (FGFR1). FP-1039 consists of the FGFR1 extracellular domain fused to the Fc domain of human IgG1. The Fc domain confers a number of favorable characteristics to FP-1039 including excellent pharmacokinetics. FP-1039 binds only to select FGFs – with the highest affinity for the “classical FGFs” that have been implicated in tumor growth. As described in our recent paper [3], FP-1039 does not bind to the hormonal FGFs. Accordingly, FP-1039 targets the “bad” FGFs that drive cancer growth and at the same time avoids inhibition of the hormonal FGFs and potential related toxicities (Fig. 1).

Several companies are targeting the FGF-FGFR signaling pathway for cancer therapeutics [4]. This includes small molecule tyrosine kinase inhibitors, which inhibit the signaling of the intracellular kinase domain; they tend to have broad specificity across FGF receptors and consequently block most FGF receptor signaling. In some instances they also inhibit other classes of receptors (e.g., VEGF receptors). Importantly these pan-FGFR inhibitors also block the activity of the hormonal FGFs which may have unwanted side effects. For example, AZD4547 has reported hyperphosphatemia in 25 of 69 treated patients in a recently reported Phase 1clinical trial [5]. Similar phosphate elevations have also been reported in preclinical toxicology studies of small molecule FGFR inhibitors [6]. These side effects may potentially be minimized by a low-phosphate diet, with drugs that remove phosphate from the blood (chelation therapy), or stopping the FGF inhibitor drug for a period of time. Other companies have tried to specifically target individual FGF receptors using monoclonal antibodies, although in some cases this has met with unexpected toxicity, as was previously shown with a monoclonal antibody directed against FGFR1 that resulted in rapid weight loss in animal models [7]. Hyperphosphatemia and weight loss have not been observed with FP-1039 [8]. While targeting individual FGF ligands may have anti-tumor efficacy in the absence of toxicity (e.g., FGF-2), FP-1039 has the advantage in that it is capable of blocking multiple FGFs that have been implicated in cancer. As such, FP-1039 has the potential for therapeutic benefit in a broader context than antibodies that targeting a single FGF.

As a result, FP-1039 is getting closer to the goal of treating the right patient with the right drug for the right target. First, FP-1039 has the ability to target and inhibit only “the classical FGFs” and not the hormonal FGFs, thus avoiding potential toxicities associated with pan-FGFR inhibition. Secondly, the direct action against a family of ligands that have been shown to promote tumor cell growth and in parallel inhibit tumor blood vessels, allows clear identification of the target and patient. Our work has shown that FP-1039 is particularly effective against tumor models that harbor amplification of the *FGFR1* gene. Such amplifications have been observed in lung cancer, including squamous non-small cell lung cancer (NSCLC) and small cell lung cancer (SCLC), as well as breast cancer. The presence of the *FGFR1* amplification may provide a biomarker to identify patients that will be particularly sensitive to therapy by FP-1039 and therefore, add to the list of drugs that offer a precision medicine approach to cancer therapy.
